# A Computational Framework for Evaluating the Efficiency of *Arabidopsis* Accessions in Response to Nitrogen Stress Reveals Important Metabolic Mechanisms

**DOI:** 10.3389/fpls.2012.00217

**Published:** 2012-09-25

**Authors:** Sabrina Kleessen, Alisdair R. Fernie, Zoran Nikoloski

**Affiliations:** ^1^Systems Biology and Mathematical Modeling Group, Max-Planck Institute of Molecular PhysiologyPotsdam, Germany; ^2^Central Metabolism Group, Max-Planck Institute of Molecular PhysiologyPotsdam, Germany

**Keywords:** data envelopment analysis, multivariate data analysis, genotypes, metabolomics, efficiency

## Abstract

High-throughput phenotyping technologies in combination with genetic variability for the plant model species *Arabidopsis thaliana* (*Arabidopsis*) offer an excellent experimental platform to reveal the effects of different gene combinations on phenotypes. These developments have been coupled with computational approaches to extract information not only from the multidimensional data, capturing various levels of biochemical organization, but also from various morphological and growth-related traits. Nevertheless, the existing methods usually focus on data aggregation which may neglect accession-specific effects. Here we argue that revealing the molecular mechanisms governing a desired set of output traits can be performed by ranking of accessions based on their efficiencies relative to all other analyzed accessions. To this end, we propose a framework for evaluating accessions via their relative efficiencies which establish a relationship between multidimensional system’s inputs and outputs from different environmental conditions. The framework combines data envelopment analysis (DEA) with a novel valency index characterizing the difference in congruence between the efficiency rankings of accessions under various conditions. We illustrate the advantages of the proposed approach for analyzing genetic variability on a publicly available data set comprising quantitative data on metabolic and morphological traits for 23 *Arabidopsis* accessions under three conditions of nitrogen availability. In addition, we extend the proposed framework to identify the set of traits displaying the highest influence on ranking based on the relative efficiencies of the considered accessions. As an outlook, we discuss how the proposed framework can be combined with well-established statistical techniques to further dissect the relationship between natural variability and metabolism.

## Introduction

Genetic variability is often deemed the most important resource for modern plant biology (see for example Koornneef et al., 2004) as it offers the means for dissecting phenotypes resulting from particular gene combinations and their effects on processes downstream of gene transcription (e.g., signaling and metabolism). Therefore, the multitude of naturally occurring genetic variability, especially for the model species *Arabidopsis*, can be used to reveal the molecular mechanisms underlying the performance of particular accessions under specific conditions. To this end, systems biology provides a unique framework to discover the reasons for any differences in molecular phenotypes (i.e., transcriptome, proteome, and metabolome) for *Arabidopsis* accessions. High-throughput phenotyping technologies in combination with accessions can then be used to gain insights into the control of cellular processes. This is of paramount importance for the study of plant growth and will likely prove crucial in efforts to ensure crop yield security.

The main problem in using molecular phenotypes in combination with growth- and yield-related morphological traits of accessions is the necessity to associate *multiple* input parameters with *multiple* system’s outputs. For instance, the cellular components participating in nitrogen uptake, assimilation, and remobilization (Masclaux-Daubresse et al., 2010) as well as in carbon assimilation and partitioning (Smith and Stitt, 2007) can be regarded as molecular inputs of plants. On the other hand, the major cellular components of a plant cell (e.g., proteins and starch), growth-related aggregated parameters (e.g., fresh weight), and system-wide morphological traits (e.g., shoot and root growth characteristics) can be treated as parts of plants’ output. Here we argue that revealing the molecular mechanisms governing a desired set of output traits can be performed by ranking of accessions based on their relative efficiencies. The latter must consider associations between multiple inputs and outputs under different environments. Further experimental efforts can in turn be focused on the molecular traits (and the related pathways) which contribute the most to the resulting condition-specific rankings.

To address the problem of determining the relative efficiency of accessions, it is desirable to combine various inputs and outputs into a single measure that adequately evaluates and ranks the “performance” of accessions represented by the use made of resources (inputs) in the attainment of outputs. An accession is efficient relative to other accessions if some of its inputs (or outputs) cannot be improved without worsening some of its other inputs (or outputs; Cooper et al., 2007). There are several approaches to determine relative efficiencies, which can be grouped into three categories: regression-based methods, dimension-reduction approaches, and optimization-based techniques. The regression-based methods generally resort to aggregation assumptions, based on parameter estimations, for the inputs and/or outputs and ultimately result in estimation of the *average (in)efficient entity* (Cubbin and Tzanidakis, 1998). Therefore, these approaches fall short in their ability to assess the reasons for the (in)efficiency specific to particular accessions. Moreover, dimension-reduction techniques, including principle component analysis (for a single data set) suffer similar problems, as they are often applied on transformed set of data consisting of input-output ratios (Zhu, 1998). In addition, more advanced techniques, such as canonical correlation analysis (for a pair of data sets), impose pre-selection of variables or data transformations to meet the assumptions, regarding the number of inputs and outputs with respect to available observations, specific to the techniques (González et al., 2008). Finally, since the results are usually expressed as relationships between linear combinations of the examined variables, the (biological) interpretability of the findings may be hampered (Tabachnick and Fidell, 1996).

In contrast, data envelopment analysis (DEA) is a non-parametric optimization technique based on linear programming for determining the relative efficiencies of entities specified by a set of inputs and outputs. DEA provides a single aggregated measure for each entity (e.g., accession) in terms of its utilization of multiple inputs to generate multiple outputs, and, therefore, its results do not pertain to *average* entity. Due to this attractive feature, DEA has found numerous application in analysis of health care efficiency, e.g., hospitals (Salinas-Jiménez and Smith, 1996; Tiemann and Schreyögg, 2012), banks (Sherman and Gold, 1985; Vassiloglou and Giokas, 1990), control of violence (Cotte Poveda, 2012), airport benchmarking (Schaar, 2008) as well as in assessing energy efficiency (Cook and Green, 2005; Azadeh et al., 2007), and economics (Charnes et al., 1989; Johnes and Johnes, 1993; Abbott and Doucouliagos, 2003). Moreover, theoretical developments (Simar and Wilson, 1999) allow for bootstrapping of the determined efficiencies and, thus, open further opportunities for applications of this technique with statistical support of the results. Nevertheless, to our knowledge, this optimization technique has not yet been applied in the analysis of molecular phenotypes of accessions.

In the following article, we provide the definition of the classical DEA and its extension, PCA-DEA, based on principal component analysis (PCA). Moreover, we propose a novel extension to PCA-DEA which can be employed to statistically validate the congruence between the rankings obtained from inputs and outputs pertaining to different environments. We also devise a valency index for each input, which can be used to pinpoint the molecular reasons for the different performance of accessions under different conditions. The computational details and outcomes of the proposed framework are illustrated by using a data set comprising the metabolomic phenotypes and morphological traits for 23 *Arabidopsis* accessions grown in three different nitrogen environments (Ikram et al., 2012). Finally, the analysis of this data set demonstrates that the framework can facilitate the biological interpretability of multidimensional data from accessions by placing the system’s components in their respective biochemical context.

## Materials and Methods

### Data envelopment analysis

Data envelopment analysis (DEA) is a computational approach, based on linear programming, which aims at determining the relative efficiency of entities, so-called decision making units (DMUs), specified with their respective inputs and outputs. In our framework, the DMUs correspond to the different *Arabidopsis* accessions. In contrast to other approaches for analysis of multidimensional data, allowing only pair-wise combination of biochemical system levels (e.g., metabolites and transcripts, or proteins and metabolites), DEA is applicable across data from multiple levels of biological organization. With the help of DEA, one can readily identify the best-performing accession by providing a ranking based on the relative efficiency. Here we extend this approach to determine the reasons (represented by particular metabolic traits) responsible for the performance of a particular accession. As opposed to other approaches for analysis of multivariate data, DEA considers all input and all output levels simultaneously. To quantitatively combine the multiple inputs and multiple outputs, DEA computes the relative efficiency of each individual accessions with respect to all other accession by employing the weighted averages, so that efficiency = weighted sum of inputs/weighted sum of outputs. While this leads to respective aggregations of inputs and outputs, we point out that, unlike in other statistical techniques for multivariate data analysis, the aggregations in DEA differ between accessions.

Consider a set of *s* accessions with each accession *a*, 1 ≤ *a* ≤ *s*, with *m* inputs $x_{i}^{a}$, 1 ≤ *i* ≤ *m*, generating *n* outputs $y_{j}^{a}$, 1 ≤ *j* ≤ *n*. The efficiency of a particular accession *a* is then given by the solution of a fractional program, originally proposed by Charnes et al. (1978):

$$\begin{array}{llll} e_{a}=\underset{\nu ,\mu }{\text{min}}\; & \frac{\overset{m}{\underset{i=1}{\sum }}\;\nu_{i}x_{i}^{a}}{\overset{n}{\underset{j=1}{\sum }}\;\mu_{j}y_{j}^{a}} & & \\ \text{subject}\;\text{to} & & & \text{(1)}\\ & \frac{\overset{m}{\underset{i=1}{\sum }}\;\nu_{i}x_{i}^{a}}{\overset{n}{\underset{j=1}{\sum }}\;\mu_{j}y_{j}^{a}}\leq 1,\;\forall a & & \\ & \nu_{i},\mu_{j}\geq 0,\;\forall i,j, & & \end{array}$$

where *v_i_* and *μ_j_* correspond to the weights associated with the input *i* and the output *j*, respectively. We point out that this model, minimizing the linear combination of inputs while producing at least the given output levels, is referred to as the *output-oriented* model. Clearly, the reciprocal of the ratio of outputs to inputs results in another type of model, named *input-oriented*, which maximizes outputs without requiring more of any of the observed inputs (Cooper et al., 2007). We note that the qualitative findings from the input- and output-oriented models, with respect to the ranking of accessions based on the relative efficiencies, are equivalent. With the help of the theory of fractional programming (Charnes and Cooper, 1962), the fractional program in equation (1) can be formulated as a linear programming (LP) problem by constraining the denominator of the objective function to one and only minimizing the numerator.

Depending on the scale assumptions in calculating the relative efficiencies, there are two basic DEA models, the CCR (Charnes, Cooper, and Rhodes) model (Charnes et al., 1978) and its extension the BCC (Banker, Charnes, and Cooper) model (Banker et al., 1984). The former formalizes the concept of constant-returns-to-scale (CRS), whereby the output changes by the same proportion as the input. On the other hand, the latter captures the concept of variable-returns-to-scale (VRS), comprising the three variants, namely: increasing-, decreasing-, and constant- returns-to-scale. Clearly, any CCR-efficient accession is also BCC-efficient. As a result, in the following, we focus on the more general BCC model to consider also the effects of increasing- and decreasing- returns-to-scale.

The LP formulation for the BCC model is given by the following:

$$\begin{array}{llll} e_{a}=\underset{\nu ,\mu ,u_{a}}{\text{min}}\; & \overset{m}{\underset{i=1}{\sum }}\;\nu_{i}x_{i}^{a}-u_{a} & & \\ \text{subject}\;\text{to} & & & \\ & \overset{n}{\underset{j=1}{\sum }}\;\mu_{j}y_{j}^{a}=1 & & \text{(2)}\\ & \overset{n}{\underset{j=1}{\sum }}\;\mu_{j}y_{j}^{a}-\overset{m}{\underset{i=1}{\sum }}\;\nu_{i}x_{i}^{a}+u^{a}\geq 0,\;\forall a & & \\ & \nu_{i},\mu_{j}\geq 0,\;\forall i,j & & \\ & u_{a}\text{unconstrained}. & & \end{array}$$

By the duality theorem, this problem is equivalent to the following LP:

$$\begin{array}{llll} e_{a}=\underset{\lambda ,s}{\max}\; & \Theta_{a} & & \\ \text{subject}\;\text{to} & & & \\ & \overset{s}{\underset{a=1}{\sum }}\;\lambda_{a}x_{i}^{a}+s_{i}=x_{i}^{a},\;\forall i & & \\ & \overset{s}{\underset{a=1}{\sum }}\;\lambda_{a}y_{j}^{a}-s_{j}=\Theta y_{j}^{a},\;\forall j & & \text{(3)}\\ & \overset{s}{\underset{a=1}{\sum }}\;\lambda_{a}=1 & & \\ & \lambda_{a},s_{i},s_{j}\geq 0,\;\forall a,i,j & & \\ & \Theta_{a}\text{unconstrained}, & & \end{array}$$

where *s_i_* and *s_j_* are the slacks of the input *i* and the output *j*, respectively, used to convert the inequalities into equivalent equations and Θ*_a_* gives the efficiency score for accession *a*. The vector *λ* represents the weights of the accessions resulting from the LP given in equation (3). By the strong duality theorem, the optimal value of the dual problem, given in equation (3), equals the optimal value of the primal problem in equation (2). The number of constraints for the primal program depends on the number of accessions, while that of the dual program depends on the number of inputs and outputs.

An accession *a* is considered (fully) BCC-efficient in the VRS sense if there exists a solution to equation (3) such that the following two conditions are satisfied:

1. Θ*_a_* = 1.
2. All slacks *s_i_*, 1 ≤ *i* ≤ *m*, and *s_j_*, 1 ≤ *j* ≤ *n*, are zero.

**Figure 1:**
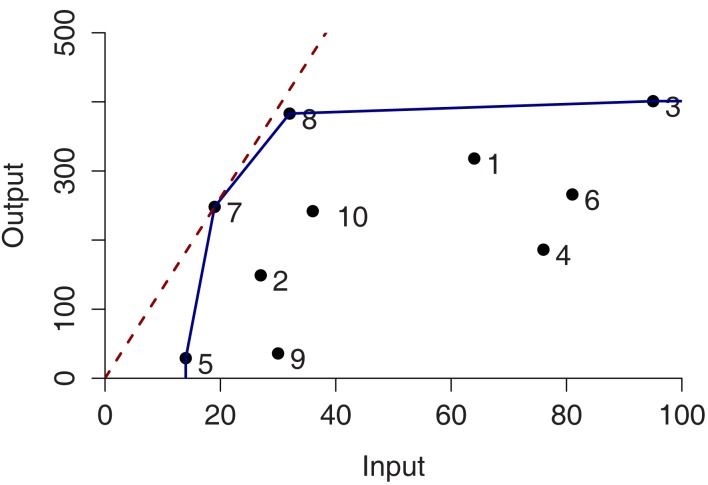
**Constant- and variable-returns-to-scale projection in the single input single output case for ten entities**. The efficient frontiers are colored in red and blue corresponding to the constant-returns-to-scale (CRS) and variable-returns-to-scale (VRS), respectively.

These two conditions define the so-called *Pareto-Koopmans efficiency*, whereby an accession is fully efficient when an attempt to improve on any of its inputs or outputs will adversely affect some other inputs or outputs. The efficient accessions define the Pareto efficiency frontier (Cooper et al., 2007). Figure 1 illustrates the efficiency frontier for the simplest case of single input and single output of 10 accessions for both CRS (red-dashed) and VRS (blue). In case of CRS, only one accession, i.e., 7, is determined as efficient whereas four accessions (i.e., 3, 5, 7, and 8) are efficient and form the Pareto efficiency frontier for the VRS.

### Combination of principal component analysis and data envelopment analysis

If the number of analyzed accessions, *s*, is less than the total number of inputs and outputs, *m* + *n*, a large number of accessions may be predicted to be efficient (depending on the structure of the data set). To resolve this issue, arising due to the multidimensionality of the data, the number of constraints imposed in the formulation of DEA in equation (2) needs to be reduced. Consequently, DEA has been combined with principal component analysis (PCA) to reduce the dimension of inputs and outputs while minimizing the loss of information (Adler and Golany, 2001).

#### Principal component analysis

Principal component analysis is a linear algebra technique which can be used to represent a set of possibly correlated variables into a set of uncorrelated variables called principal components (PCs). Each principal component is represented as a linear combination of the original variables. The coefficients in the linear combination are given by the eigenvectors from the eigenvalue decomposition of the covariance matrix for the analyzed set of variables. The PCs are usually ordered by the percentage of the accounted variance, starting with the component of the largest variance. It should be noted, that the number of PCs is less than or equal to the number of original variables (Abdi and Williams, 2010).

The variance ζ*_k_* (1 ≤ *k* ≤ *K*) explained by the *k*-th PC is calculated as: $\zeta_{k}=\alpha_{k}∕\sum_{l=1}^{K}\;\alpha_{l}$, where *K* is the number of original variables and α*_l_* (1 ≤ *l* ≤ *K*) is the *l*-th largest eigenvalue of the covariance matrix for the *K* variables. The number of PCs used in analyses depends on the percentage of variance to be explained for a particular purpose (over 80% is a common choice in applications). Indeed, considering all PCs amounts to using the original data set.

#### Formulation of PCA-DEA

The usage of PCs instead of the original data induces a transformation of the DEA model. Therefore, the inputs *X* and the outputs *Y* are transformed through PCA.

Let $L_{k}^{X}$ and $L_{k}^{Y}$ denote the matrices containing the coefficients of the linear combinations rendering the PCs of the input and output data, respectively. The size of this matrix is reduced to the number of PCs which explain a pre-specified percentage of the variance in the original data. Then, $X_{k}=L_{k}^{X}X$ and $Y_{k}=L_{k}^{Y}Y$ are the *k* PCs, i.e., linear combinations, of the variables in the data sets *X* and *Y*. Furthermore, the number of columns in *X_k_* and *Y_k_* correspond to the number *k* of PCs used to represent the input and output data.

Consequently, the general BCC model from equation (2) for accession *a* can be transformed as follows:

$$\begin{array}{llll} e_{a}=\underset{\mathrm{V,U,}u_{a}}{\text{min}}\; & V_{k}X_{k}^{a}-u_{a} & & \\ \text{subject}\;\text{to} & & & \\ & U_{k}Y_{k}^{a}=1 & & \\ & U_{k}Y_{k}-V_{k}X_{k}+u_{a}\geq 0 & & \text{(4)}\\ & V_{k}L_{k}^{X}\geq 0 & & \\ & U_{k}L_{k}^{Y}\geq 0 & & \\ & V_{k},U_{k},u_{a}\text{unconstrained}, & & \end{array}$$

where *U_k_* and *V_k_* denote for the coefficients of the PCs used for the input and output data. Since $V_{k}X_{k}=V_{k}L_{k}^{X}X$, where *V_k_* represents a row vector of dual variables, the weights of the original input *X* can be expressed as $V_{k}L_{k}^{X}$. We note that the same holds for the output *Y*.

Furthermore, the corresponding dual program can be rewritten as follows:

(5) $$\begin{array}{l} \Theta_{a}=\underset{\lambda ,s}{\max}\Theta \\ \text{ subject to}\\ \;X_{k}\lambda +L_{k}^{X}s_{k}^{X}=X_{k}^{a}\\ \;Y_{k}\lambda -L_{k}^{Y}s_{k}^{Y}=\Theta Y_{k}^{a}\\ \;\sum \lambda =1\\ \;\Theta ,\lambda ,s_{k}^{X},s_{k}^{Y}\geq 0. \end{array}$$

The problem in equation (5) is referred to as the *envelopment* problem. Like the primal program given in equation (4), it provides weights for each accession, indicating those accession of highest influence to the efficiency of the accession *a* for which the efficiency is calculated.

### Statistical analysis

The Kendall rank correlation coefficient, denoted by *τ*, evaluates the degree of similarity between two sets of ranks over the same set of objects (Abdi, 2007). It is determined by the following expression:

$$\tau =\frac{\text{number}\;\text{of}\;\text{concordant}\;\text{pairs}-\text{number}\;\text{of}\;\text{discordant}\;\text{pairs}}{\text{total}\;\text{number}\;\text{of}\;\text{pairs}},$$

where a pair of ranked sets (*x_i_*, *y_i_*) and (*x_j_*, *y_j_*) (on the same set of objects) is concordant if the order of both objects agree, i.e., if both *x_i_* > *x_j_* and *y_i_* > *y_j_* or *x_i_* < *x_j_* and *y_i_* < *y_j_*. In contrast, a pair is discordant if *x_i_* < *x_j_* and *y_i_* > *y_j_* or if *x_i_* > *x_j_* and *y_i_* < *y_j_*. If *x_i_* = *x_j_* or *y_i_* = *y_j_*, the pair is neither concordant nor discordant. Larger (positive) values of *τ* indicate a greater agreement between the two sets, while smaller (negative) values imply disagreement.

We use the Kendall rank coefficient in order to capture the effect of a particular input on the resulting ranking of the accessions based on PCA-DEA. To this end, the influence of an input parameter *t* on the relative efficiency of a given accession under a condition *c* is determined by excluding *t* from the inputs and applying PCA-DEA to obtain the efficiencies $e_{c}^{-t}$ under condition *c*. We then use Kendall’s *τ* to qualitatively discriminate between different inputs (i.e., metabolic traits) with respect to their correspondence to the obtained efficiencies, resulting in:

$$\tau_{c}^{t}=\tau \left(e_{c},e_{c}^{-t}\right),$$

where *e_c_* are the efficiencies including all inputs.

Furthermore, we propose a *valency index*, denoted by *Φ*, for a metabolic trait *t* between two conditions *c*_1_ and *c*_2_. We define the valency index as the absolute value of difference between the Kendall *τ* of the efficiencies for the two conditions *c*_1_ and *c*_2_ with and without the particular metabolic trait *t*. More formally, the valency *Φ* for a metabolic trait *t* is given by:

$$\phi_{c_{1},c_{2}}^{t}=|\tau \left(e_{c_{1}},e_{c_{2}}\right)-\tau \left(e_{c_{1}}^{-t},e_{c_{2}}^{-t}\right)|.$$

### Implementation

All mathematical programming approaches are implemented in MATLAB 7.8.0, R2009a with the optimization platform TOMLAB v7.6 (Holmström, 1999). We use CPLEX to solve the considered LP problems.

### Data set

Plant growth data comprising morphological traits and metabolic parameters are obtained from a study by Ikram et al. (2012), where natural variability of *Arabidopsis thaliana* was experimentally analyzed. The data set includes the following 23 accessions: Akita, Alc-0, Bay-0, Bl-1, Blh-1, Bur-0, Can-0, Col-0, Ct-1, Cvi-0, Edi-0, Ge-0, Gre-0, Jea, Kn-0, Ler, Mh-1, Mt-0, N13, Oy-0, Pyl-1, Sakata, and Stw-0.

In order to characterize developmental variations among the accessions in response to different nitrogen (N) supplies [i.e., normal (N+), N limited (N−), and N starved (N0)], morphological traits that contribute to plant growth were investigated. To estimate plant growth leaf number (LN) was counted, shoot projected area (SPA) was estimated from images of plants taken at 35 days of growth, shoot growth rate (SGR) was computed from rosette area measurements at 33–35 days of growth, and shoot fresh matter (SFM) was weighed at harvesting. In addition, variations of root growth were investigated by studying complementary traits, root fresh matter (RFM), primary root length (PRL) at harvesting, and the ratio of RFM to its respective PRL, termed root thickness (RT).

For the sake of characterizing N and carbon (C) metabolism, shoot and root nitrate contents (denoted by SNO3 and RNO3, respectively), shoot and root amino acid contents (SAA and RAA, respectively), shoot total nitrogen percentage (SN%), and starch contents in shoot and root (SStarch and RStarch) were measured. Finally, ratios were calculated to estimate allocation between the shoot and root during growth. The shoot to root fresh matter ratio (S/RFM) provided information about biomass allocation, whereas the ratios of SAA to RAA (S/RAA), SNO3 to RNO3 (S/RNO3), and SStarch to RStarch (S/RStarch) reflected the differences in N and C partitioning (Ikram et al., 2012).

The proposed framework is applied to identify the set of metabolic traits having the highest influence on the efficiency ranking of the considered accessions, therefore, the metabolic traits (SNO3, RNO3, SAA, RAA, SN%, SStarch, RStarch, S/RAA, S/RNO3, and S/RStarch) are included as inputs whereas the morphological traits (LN, SPA, SGR, SFM, RFM, S/RFM, PRL, and RT) are treated as the corresponding outputs (product traits).

## Results and Discussion

Here we present the results of applying the proposed framework on the data set of 23 *Arabidopsis* accessions, with 10 inputs and 8 outputs, described in Materials and Methods. To demonstrate the robustness of the findings when using principal components explaining a different percentage of the variance, we conduct comparative analysis of the findings between the three cases corresponding to at least 85, 90, and 95% variance explained, respectively.

For the inputs, 85% of the variance is explained by the first 5 principal components, while for the outputs, by the first 4 principal components. By invoking the LP in equation (5), we next determine the relative efficiencies for the accessions. The number of fully efficient accessions (i.e., whose relative efficiency is 1) is 16, 8, and 11 for the nitrogen starved (N0), nitrogen limited (N−), and the normal nitrogen (N+) condition, respectively, as summarized in Table 1. Seven of the accessions are determined as fully efficient across the three nitrogen supply conditions, named: Bur-0, Edi-0, Ge-0, Mh-1, Oy-0, Pyl-1, and Stw-0 (see Table 2). By calculating the Kendall *τ* coefficient between efficiencies of the accessions for all pairs of conditions, we confirm that the accessions under the N− condition perform more similarly to the N+ condition (τ = 0.42, *p*-value < 0.05) in comparison to the N0 (*τ* = 0.30). Moreover, as shown in Table 1, the concordance between N0 and N+ is as good as that between N− and N+, indicating that considerable metabolic responses shape the changing phenotypic traits under the two extreme conditions.

**Table 1:** **Comparative analysis of the results from PCA-DEA with 85, 90, and 95% of variance explained**.

	N+	N−	N0	No. FEff.
**85%**
N+	1	0.42*	0.44*	11
N−	0.42*	1	0.30	8
N0	0.44*	0.30	1	16
**90%**				
N+	1	0.16	0.13	17
N−	0.16	1	−0.07	11
N0	0.13	−0.07	1	20
**95%**				
N+	1	0.18	0.19	18
N−	0.18	1	0.05	16
N0	0.19	0.05	1	20

The Kendall *τ* coefficient between the relative efficiencies for the 23 accessions are presented in columns 1–3. The statistically significant correlation at level 0.05 are marked with *. The number of fully efficient accessions for the three considered conditions (N+, N−, and N0) are included in column 4, named No. FEff.

**Table 2:** **Efficiency values for the accessions obtained from PCA-DEA with 85, 90, and 95% of variance explained**.

	85%			90%			95%		
	N+	N−	N0	N+	N−	N0	N+	N−	N0
Akita	1.062	1.127	1.061	**1.000**	1.112	**1.000**	**1.000**	**1.000**	**1.000**
Alc-0	1.087	1.240	1.090	1.083	1.045	**1.000**	1.045	1.011	**1.000**
Bay-0	1.049	1.046	**1.000**	**1.000**	1.034	**1.000**	**1.000**	1.010	**1.000**
BL-1	1.015	1.103	**1.000**	1.011	1.054	**1.000**	**1.000**	1.033	**1.000**
Blh-1	1.103	1.231	**1.000**	1.082	1.145	**1.000**	1.030	1.089	**1.000**
Bur-0	**1.000**	**1.000**	**1.000**	**1.000**	**1.000**	**1.000**	**1.000**	**1.000**	**1.000**
Can-0	1.012	1.044	**1.000**	**1.000**	**1.000**	**1.000**	**1.000**	**1.000**	**1.000**
col-0	**1.000**	1.143	**1.000**	**1.000**	1.135	**1.000**	**1.000**	**1.000**	**1.000**
Ct-I	**1.000**	1.131	1.093	**1.000**	1.081	1.003	**1.000**	**1.000**	1.003
Cvi-0	1.135	1.017	1.286	1.062	**1.000**	1.170	1.050	**1.000**	1.170
Edi-0	**1.000**	**1.000**	**1.000**	**1.000**	**1.000**	**1.000**	**1.000**	**1.000**	**1.000**
Ge-0	**1.000**	**1.000**	**1.000**	**1.000**	**1.000**	**1.000**	**1.000**	**1.000**	**1.000**
Gre-0	1.065	**1.000**	**1.000**	1.063	**1.000**	**1.000**	1.062	**1.000**	**1.000**
Jea	1.072	1.284	**1.000**	1.062	1.097	**1.000**	1.038	1.089	**1.000**
Kn-0	**1.000**	1.141	**1.000**	**1.000**	1.118	**1.000**	**1.000**	1.026	**1.000**
ler	1.099	1.176	1.071	**1.000**	1.058	**1.000**	**1.000**	**1.000**	**1.000**
Mh-1	**1.000**	**1.000**	**1.000**	**1.000**	**1.000**	**1.000**	**1.000**	**1.000**	**1.000**
Mt-0	**1.000**	1.062	**1.000**	**1.000**	1.062	**1.000**	**1.000**	**1.000**	**1.000**
N13	1.021	1.057	1.242	**1.000**	**1.000**	1.184	**1.000**	**1.000**	1.184
Oy-0	**1.000**	**1.000**	**1.000**	**1.000**	**1.000**	**1.000**	**1.000**	**1.000**	**1.000**
pyl-1	**1.000**	**1.000**	**1.000**	**1.000**	**1.000**	**1.000**	**1.000**	**1.000**	**1.000**
sakat	1.022	1.248	1.005	**1.000**	1.207	**1.000**	**1.000**	1.163	**1.000**
stw-0	**1.000**	**1.000**	**1.000**	**1.000**	**1.000**	**1.000**	**1.000**	**1.000**	**1.000**

*The efficiency values for the different accessions under the three nitrogen supplies as well as three different percentages of variance explained are presented. An accession with efficiency value 1 is fully efficient (marked in bold), whereas higher values indicate poorer performance*.

For comparison, 90% of the variance is explained by the first 5–6 and 4–5 principal components for the inputs and outputs, respectively, depending on the analyzed condition. The number of fully efficient accessions is 20, 11, and 17 for the N0, N−, and N+ conditions, respectively, as summarized in Tables 1 and 2. Eight of the accessions are determined as fully efficient across the three nitrogen supply conditions, including the seven found for the case of 85% variance and in addition Can-0, revealing a close agreement between the results for the slightly different data. By calculating the Kendall *τ* coefficient with the efficiencies for each pair of conditions, we reconfirm the expectation that the similarity between the N− and N+ conditions (*τ* = 0.16) coincide with that of N0 and N+ (see Table 1). In general, the efficiency obtained from PCA-DEA for 85, 90, and 95% variance shown in Table 2 indeed confirm the robustness of the method especially with respect to the accessions predicted to be efficient.

**Figure 2:**
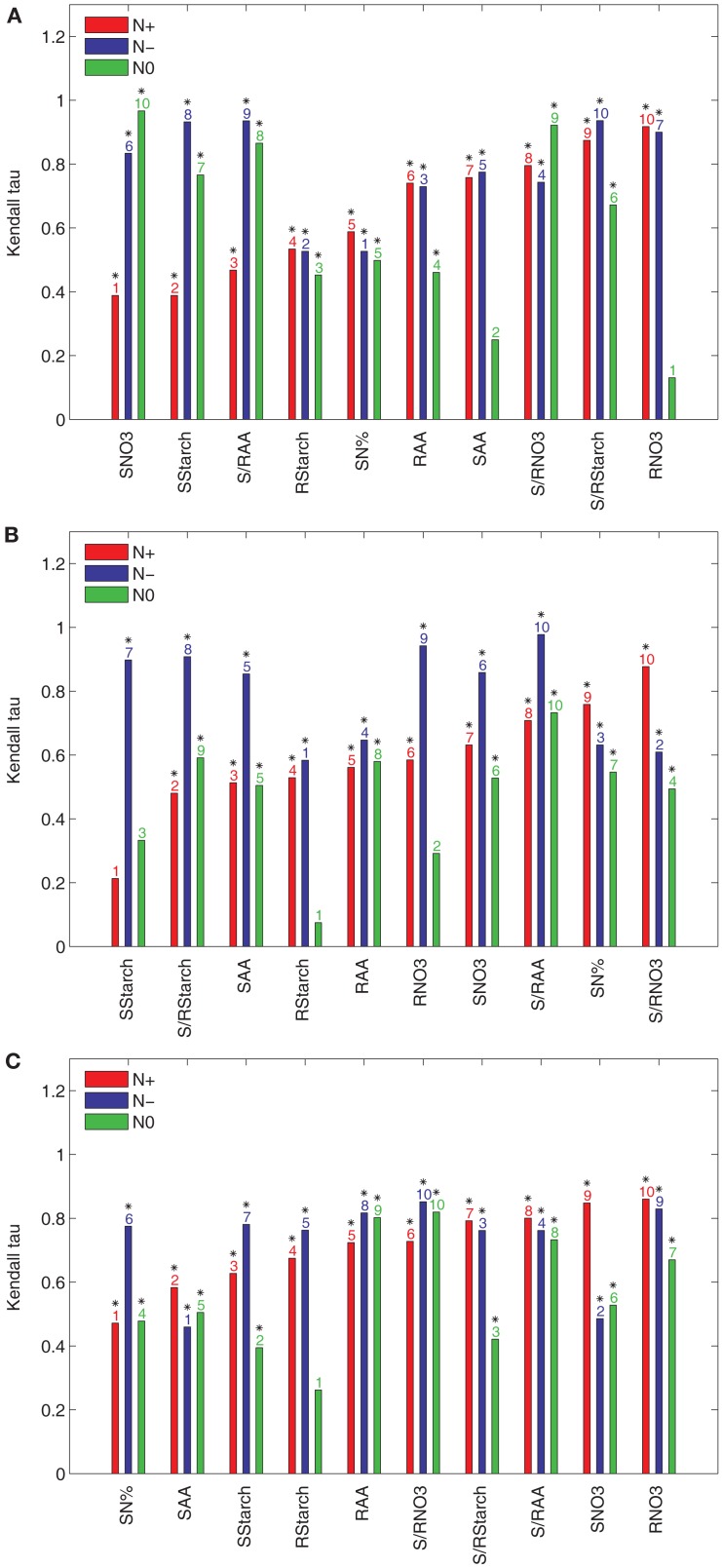
**Kendall *τ* of the results from PCA-DEA with 85, 90, and 95% of variance explained**. The Kendall *τ* coefficients between the rankings of the relative efficiencies for the 23 accessions are obtained from the data sets of the three considered conditions (N+, N−, and N0) with and without a metabolic trait *t* indicated in the *x*-axis of each panel when the principal components explaining **(A)** 85%, **(B)** 90%, and **(C)** 95% of the variance are used. The metabolic traits are sorted in an ascending order of the respective Kendall *τ* coefficients with respect to their effect in the N+ condition. The *y*-axis shows the Kendall *τ* coefficients belonging to a particular metabolic trait under normal nitrogen (N+, red), limited nitrogen (N−, blue), and starved nitrogen (N0, green) supply. The ordering of metabolic traits for the different nitrogen availabilities is indicated above the bars. Significant Kendall *τ* correlation (*p*-value < 0.05) is marked with a black star above the bar numbers.

To quantify the effect of an individual input *t* to the ranking of accessions based on their relative efficiencies, we determine the Kendall *τ* correlation between the rankings obtained from the data set with and without the input *t*. The results obtained with the principal components explaining 85, 90, and 95% of the variance are presented in Figure 2 for the N+, N−, and N0 conditions. The inputs are sorted in an increasing order with respect to their effect in the N+ condition. Clearly, the input variable which has a high effect is expected to increase the discordance between the ranking of accessions, i.e., the value of the Kendall *τ* is closer to zero.

The results in Figure 2A indicate that the nitrate and starch contents in the shoot (SNO3 and SStarch, respectively), have the largest effect on the ranking, followed by the ratio of amino acid content between the shoot and root (S/RAA) and the starch content in the root (RStarch). The only input variable shared between the three conditions is RStarch under normal nitrogen supply (N+) when the principal components explaining 85% of the variance are used. Interestingly, the nitrate content in the root (RNO3) has the smallest effect. As shown in Figure 2A the total nitrogen content in the shoot (SN%) and the starch content in the root (RStarch) have the largest effect for the N− condition, followed by the amino acid content in the root (RAA) and the ratio of the ratio of the nitrate content between the shoot and the root (S/RNO3). Finally, Figure 2A points out that, for the N0 condition, the nitrate content in the root (RNO3) has the largest effect, followed by the amino acid content in the shoot (SAA), Rstarch, and RAA. By comparing these results with those obtained from the principal components explaining 90 and 95% of the variance, presented in Figures 2B,C, one indeed confirms the robustness of the findings.

**Figure 3:**
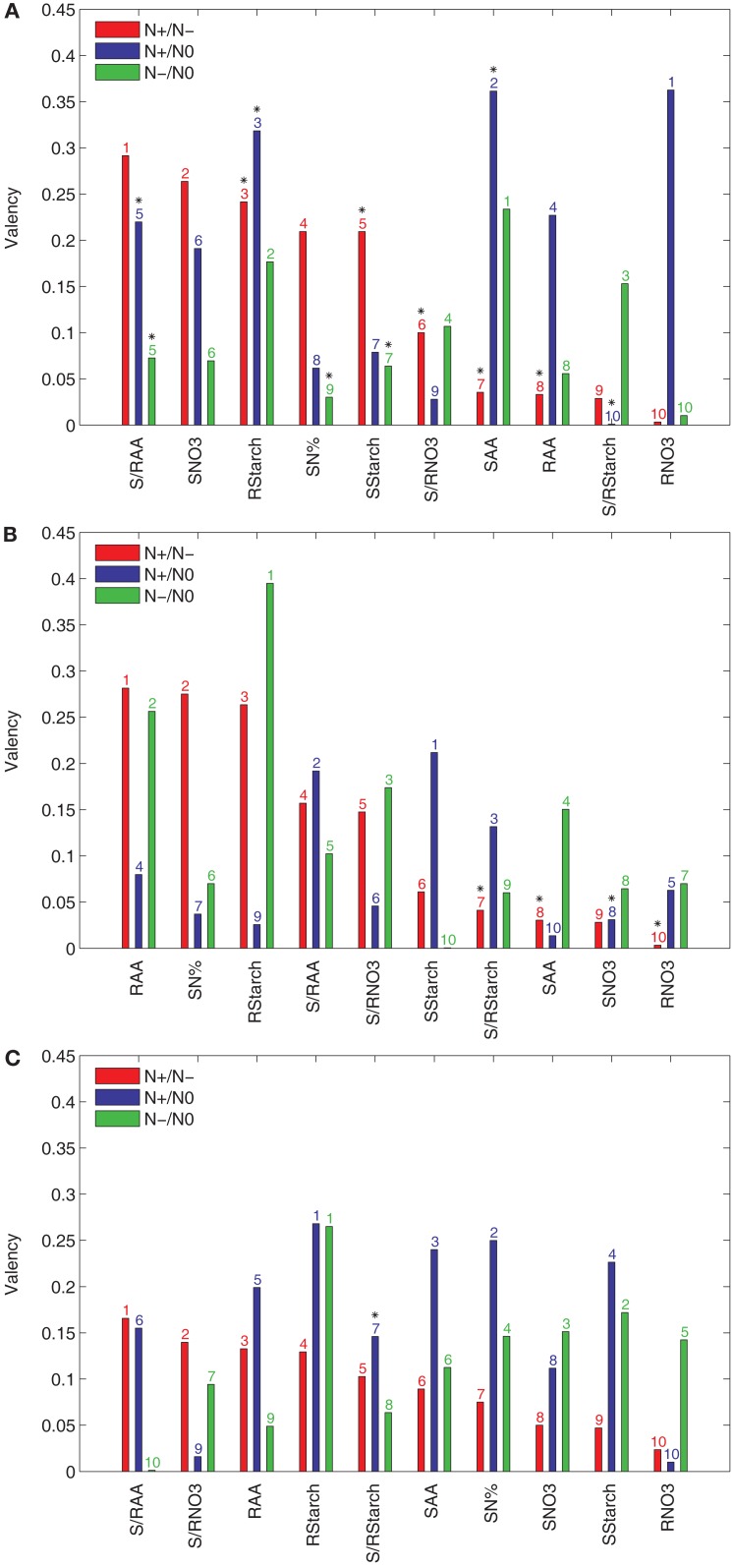
**Valency index *Φ* of the results from PCA-DEA with 85, 90, and 95% of variance explained**. The valency index between the rankings of the relative efficiencies for the 23 accessions are obtained from the pair-wise comparison between the three considered conditions (N+, N−, and N0) with and without a metabolic trait *t* indicated in the *x*-axis of each panel when the principal components explaining **(A)** 85%, **(B)** 90%, and **(C)** 95% of the variance are used. The metabolic traits are sorted in a descending order of their valencies with respect to their effect in the comparison of N+ and N−. The *y*-axis refers to the valency index (*Φ*) of a particular metabolic trait in the comparative analysis of N+ and N− (red), N+ and N0 (blue), and N− and N0 (green) conditions. The ordering of metabolic traits for the different nitrogen availabilities is indicated above the bars. Significant Kendall is *τ* correlation (*p*-value < 0.05) is marked with a black star above the bar numbers.

The importance of an input variable for the concordance of accession rankings between two conditions can be estimated by the proposed valency index. It is easy to interpret the valency of an input *t*, since a larger value indicates a larger effect of the input with respect to the behavior between the two conditions. In such a way, genes and gene interactions acting on the pathways in the vicinity of the input variables of high valency may hold the answer for explaining the diverse responses due to the natural variability. In contrast to Figure 2, here the input variables are sorted in a decreasing order of their valencies with respect to their effect by comparing N+ and N− conditions (see Figure 3).

The results in Figure 3A indicate that the S/RAA, SNO3, and RStarch have the highest valencies when the principal components explaining 85% of the variance are used in the comparative analysis of the N+ and N− conditions. These were followed by SN% and SStarch, obtaining a valency value of 0.2. On the other hand, RNO3 and SAA are of largest valency when the N+ and N0 conditions are compared. When N− and N0 are compared, the highest valency is obtained for SAA. The next two inputs, namely RStarch and S/RStarch, already have a value below 0.2 for the valency. Moreover, this suggests that the largest portion of the difference between accession performances under changing nitrogen supply may be due to natural variability in the genes controlling the supply of nitrate to or amino acid metabolism within the shoot.

These conclusions are in close accordance with what has been experimentally established in a wide range of plant species, namely, that carbon and nitrogen metabolism are highly intertwined (Stitt and Fernie, 2003; Nunes-Nesi et al., 2010). The finding that shoot amino acid content is the trait contributing most to the discordance in rankings between the various nutrient conditions is in accordance with the fact that in *Arabidopsis*, most nitrate taken up by root transporters is reduced either in the roots or shoots but assimilated into amino acids predominantly in the shoots (Masclaux-Daubresse et al., 2010). The importance of starch levels both in the root and the shoot for this efficiency is also in keeping with its recent definition, on the basis of a much larger screen than that of the Ikram et al. (2012) study, as a major integrator or metabolism and growth (Sulpice et al., 2009) as well as observations that nitrate acts as a signal to repress starch synthesis in a range of species (Scheible et al., 1997; Nunes-Nesi et al., 2010). For completeness, the valencies resulting from principal components that account for 90 and 95% of the variance are shown in Figures 3B,C.

The same data set was analyzed in the original publication with the help of classical statistical methods (i.e., ANOVA), hierarchical clustering, and descriptive network-based analysis (Ikram et al., 2012). By applying ANOVA, the authors determined the effect in each input due to the two factors (N conditions and the accessions) and their interaction, as percentage of explained variation. In such a way, however, the authors could elicit trends over all accessions in a single condition, but not between conditions. Moreover, the Pearson correlation analysis was carried out on the profiles for each input/output over all accessions. In such a way, the individual differences between accessions may again be neglected. Nevertheless, the authors found that, in the N+ condition, S/RNO3 is the trait with the largest number of significant correlations to the other traits. In the N− condition these included RFM (product trait) and RAA (input trait), while in the N0 condition these comprised the two product traits SFM and RT. While S/RNO3 may offer some glimpses in the mechanisms for the N+ condition, these cannot be obtained for the other two conditions, since morphological traits, far away from metabolism, are suggested to be of importance. This is the reason, perhaps, why the authors considered SAA and RAA as the input traits which correlate, albeit poorly, with SFM and RT in the N0 condition. Finally, the authors conducted a clustering analysis of the accessions but now with the profiles of traits over the three conditions. Although the clustering distinguished four classes of accessions, this finding can hardly be related to biochemical mechanisms since the specificity of inputs and output traits is lost due to the consideration of the data from all conditions at once.

## Outlook and Conclusion

Predictions of the global population over the next 50 years suggest that crop productivity needs to be massively improved. In parallel, current environmental deterioration is reducing crop yields and decreasing the availability of arable land. Against these twin facts a major challenge of agriculture is to enhance plant productivity whilst at the same time minimize additional costly inputs such as fertilizers and plant protectants. Reduction of such inputs, in the case study used specifically to increase the nitrate use efficiency, would likely additionally be beneficial from an environmental perspective.

In this study, we propose a framework based on PCA-DEA combining multiple inputs and outputs into a single measure, which can then be employed to statistically validate the performance of accessions under various conditions. In addition, we extend this approach to not only identify the ranking, but also the set of traits which has the highest influence on the efficiency ranking of the considered accessions for a single condition and pairs of conditions. By using the proposed framework with the recently published data set of Ikram et al. (2012) we were able to pinpoint the most important metabolic traits with respect to the overall growth efficiency under varying environmental conditions. Additional experimental validation would be necessary to confirm the plausible causal role of these metabolic traits with respect to efficiency under varying conditions.

However, we believe that this is just one possible application of DEA to analyze heterogeneous data from various (high-throughput) phenotyping technologies. The separation between classes of entities (e.g., accessions) based on the relative efficiencies, indicates the possibility of using (PCA)-DEA as a data preprocessing/separation technique. Other statistical techniques can then be used on the classes of relative efficiencies resulting from DEA. It is our contention that this approach will prove to be a generally applicable aid in rational design strategies for crop metabolic engineering.

## Conflict of Interest Statement

The authors declare that the research was conducted in the absence of any commercial or financial relationships that could be construed as a potential conflict of interest.
